# Supplementary material for: Empirical methods for controlling false positives and estimating confidence in ChIP-Seq peaks
Source: BMC Bioinformatics. 2008 Dec 5;9:523. doi: 10.1186/1471-2105-9-523 (PMC2628906; doi:10.1186/1471-2105-9-523)
Supplement: Additional File 1 — A variety of html documents from the USeq web site detailing the available applications, their best usage, output file type descriptions, command line menus, etc. [file 1471-2105-9-523-S1.zip › USeqUserGuides/JavaDoc.zip!/JavaDoc/util/bio/annotation/package-frame.html]

util.bio.annotation


util.bio.annotation

Classes 
  
Coordinate
  
ExonIntron
  
ExportExons
  
ExportIntergenicRegions
  
ExportIntronicRegions
  
GeneGroup
  
GeneRep
  
GenericFeature
  
ScoredCoordinate
  
StartStop
  
Transcript
  
TransGroup
  
Translation
